# The Private Clinic

**Published:** 1898-03-26

**Authors:** 


### THE PRIVATE CLINIC.

The private hospital, not the pay hospital to which any medical man can send his case and at which he can continue his attendance, but the hospital which is the private venture of the medical man, is as yet in its infancy in England. Of course such things exist, and to those who possess the faculty of organisation they afford both profit and fame. These institutions, however, have never become established as clinics as well as hospitals in the open way that is common in other countries. They have always been rather allied to what are called nursing homes; and, as nursing homes have increased in number and improved in fittings and efficiency, the tendency has drifted of late years in the direction of surgeons treating their cases in institutions run and perhaps owned by nurses rather than in hospitals of their own. Trouble is doubtless avoided, while dignity is certainly saved by not combining fees for operation with those for food and lodging. Abroad, however, neither doctors or patients are so squeamish, and thus it happens that we find handsome private hospitals which are teaching hospitals as well, institutions from which their medical owners derive both money and reputation—money from the operations and the board and lodging, and professional repute from the teaching given in their clinics.

An American physician, Dr. Preston Miller, has been making a tour of Europe, spending many months therein, and learning all he could, and in giving some of his impressions to the *Medical Record* he speaks of Berlin as a city of private clinics for women's diseases. These, he says, are often fine properties, built and possessed by the most celebrated gynæcologists, who have great wealth and derive large incomes from their practice. He describes Landow's clinic as being under the control of the owner and a younger brother. It is evidently a large establishment, with operating room, anæsthetic room, and an adjoining room for preparing the person of the operator; but the point to which we wish to draw attention is that it is not conducted, like an English "nursing home," in such a manner as to make the patient feel as nearly as possible as if at home, but is worked on the principle of a hospital. "On the same floor is a very large room with seats for quite a large class, and gynæcological chairs and appliances. Here examinations and diagnoses are made, tampons and pessaries applied, and trivial operations sometimes done." Here come physicians and gynæcologists from all parts of the world. Teaching is the order of the day. There is an extensive laboratory, where pathology, bacteriology, and histology are ably taught by the first assistant, who is described as perhaps the speediest pathologist in Germany. This gentleman, it appears, was seen to freeze specimens of new growths, and cut, stain, and examine them under the microscope in seven minutes, while the operation was in progress, while, in fact, the pelvis was open and the operator was in doubt how far he should go until he could be assured as to the malignancy or otherwise of the tumour.

It is the class that makes the reputation, and when medical men in London not only own their hospitals, but also use them for teaching purposes, we shall doubtless see a very radical change in many professional usages in this country.

